# Cortical Correlates of Psychedelic-Induced Shaking Behavior Revealed by Voltage Imaging

**DOI:** 10.3390/ijms24119463

**Published:** 2023-05-30

**Authors:** Tobias Buchborn, Taylor Lyons, Chenchen Song, Amanda Feilding, Thomas Knöpfel

**Affiliations:** 1 Laboratory for Neuronal Circuit Dynamics, Imperial College London, London W12 0NN, UK; t.lyons15@imperial.ac.uk (T.L.); song@knopfel-lab.net (C.S.); tknopfel@knopfel-lab.net (T.K.); 2 Institute of Psychopharmacology, Central Institute of Mental Health, Medical Faculty Mannheim, University of Heidelberg, 69115 Heidelberg, Germany; 3 Centre for Psychedelic Research, Department of Medicine, Imperial College London, London W12 0NN, UK; 4 The Beckley Foundation, Beckley Park, Oxford OX3 9SY, UK; amanda@beckleyfoundation.org; 5 Centre for Neurotechnology, Institute of Biomedical Engineering, Imperial College London, London SW7 2AZ, UK; 6 Laboratory for Neuronal Circuit Dynamics, Hong Kong Baptist University, Kowloon Tong, Hong Kong

**Keywords:** voltage imaging, pyramidal cells, psychedelic, 5-HT2A receptor, wet dog shakes, hemodynamics, genetically encoded voltage indicator (GEVI)

## Abstract

(1) From mouse to man, shaking behavior (head twitches and/or wet dog shakes) is a reliable readout of psychedelic drug action. Shaking behavior like psychedelia is thought to be mediated by serotonin 2A receptors on cortical pyramidal cells. The involvement of pyramidal cells in psychedelic-induced shaking behavior remains hypothetical, though, as experimental in vivo evidence is limited. (2) Here, we use cell type-specific voltage imaging in awake mice to address this issue. We intersectionally express the genetically encoded voltage indicator VSFP Butterfly 1.2 in layer 2/3 pyramidal neurons. We simultaneously capture cortical hemodynamics and cell type-specific voltage activity while mice display psychedelic shaking behavior. (3) Shaking behavior is preceded by high-frequency oscillations and overlaps with low-frequency oscillations in the motor cortex. Oscillations spectrally mirror the rhythmics of shaking behavior and reflect layer 2/3 pyramidal cell activity complemented by hemodynamics. (4) Our results reveal a clear cortical fingerprint of serotonin-2A-receptor-mediated shaking behavior and open a promising methodological avenue relating a cross-mammalian psychedelic effect to cell-type specific brain dynamics.

## 1. Introduction

Serotonergic psychedelics, such as lysergic acid diethylamide (LSD), profoundly affect human psychological functioning. In rodents, psychedelics induce stereotypical motor behaviors, including backward walking, reciprocal forepaw treading, flat body posture, lateral head weaving, and/or head twitches, and wet dog shakes. The last two behavioral components, hereon together referred to as shaking behavior [1,2], rank among the most widely used animal behavioral correlates of central serotonin activity. As an animal model of neuropsychiatric conditions, shaking behavior is a particularly appealing behavioral readout. In mammals, shaking behavior is innate and has a benign character already infrequently exhibited as a part of the natural repertoire, readily observable by eye, and particularly targets one constituent of serotonin transmission, namely the serotonin (5-HT) 2A receptor. Psychedelic-induced shaking behavior across species has been described from as early as 1956 [3,4,5]. Correlation studies showed a close relationship between the potency of diverse antagonists to block shaking behavior and their affinity for 5-HT2A receptors [6,7,8]. Further, the importance of 5-HT2A receptors in shaking behavior has recently been reaffirmed using a 5-HT2A receptor knock-out mouse model [9,10]. Despite half a century of research, our understanding of the function and physiology of this behavioral stereotype remains limited. 5-HT2A receptors are most abundantly expressed in the cerebral cortex, and tolerance to shaking behavior has been shown to reflect adaptation in 5-HT2A signaling and/or binding sites in the (frontal) cortex [1,11]. Further, the inability to display shaking behavior in 5-HT2A receptor knock-out mice is reversed by selective restoration of 5-HT2A receptor expression in cortical pyramidal neurons [9]. Despite these and other findings collectively pointing to a possible role of cortical pyramidal neurons in the generation and/or modulation of shaking behavior under the influence of 5-HT2A receptor signaling [12], this remains controversial due to inconsistencies in the literature [13,14] and the methodological difficulties in cell type-specific measurement from awake animals.

To the best of our knowledge, so far there are only two papers that report on event-related electrophysiology of rodent shaking behavior in vivo. Neither of them has provided a cell-type-specific resolution [15,16]. Here, we address this unknown by taking advantage of recent developments in cell-type-specific voltage imaging approaches using genetically encoded voltage indicators (GEVIs) [17]. Research on the cortical effects of psychedelics is generally focused on pyramidal cells of layer 5 [12,18]. Layer 2/3 pyramidal cells—despite being major drivers of layer 5 [19]—are largely ignored. We selectively expressed the GEVI VSFP Butterfly 1.2 in cortical layer 2/3 pyramidal neurons [20], a cell population sensitive to psychedelics [21,22,23], to investigate the brain activity associated with the shaking behavior induced by the selective 5-HT2A receptor agonist 25CN-NBOH (N-(2-hydroxybenzyl)-2,5-dimethoxy-4-cyanophenylethylamine) [24]. As 5-HT2A receptor expression is not restricted to neurons but also extends across the vascular system [25,26], we additionally take advantage of the dual-emission design of VSFP Butterfly 1.2 [27] to delineate both voltage activity for cortical pyramidal neurons as well as blood-volume related hemodynamics associated with shaking behavior.

## 2. Results

### 2.1. 25CN-NBOH Altered Blood-Volume Related Signal Oscillations (BvSOs) in the Motor Cortex

**Figure 1:**
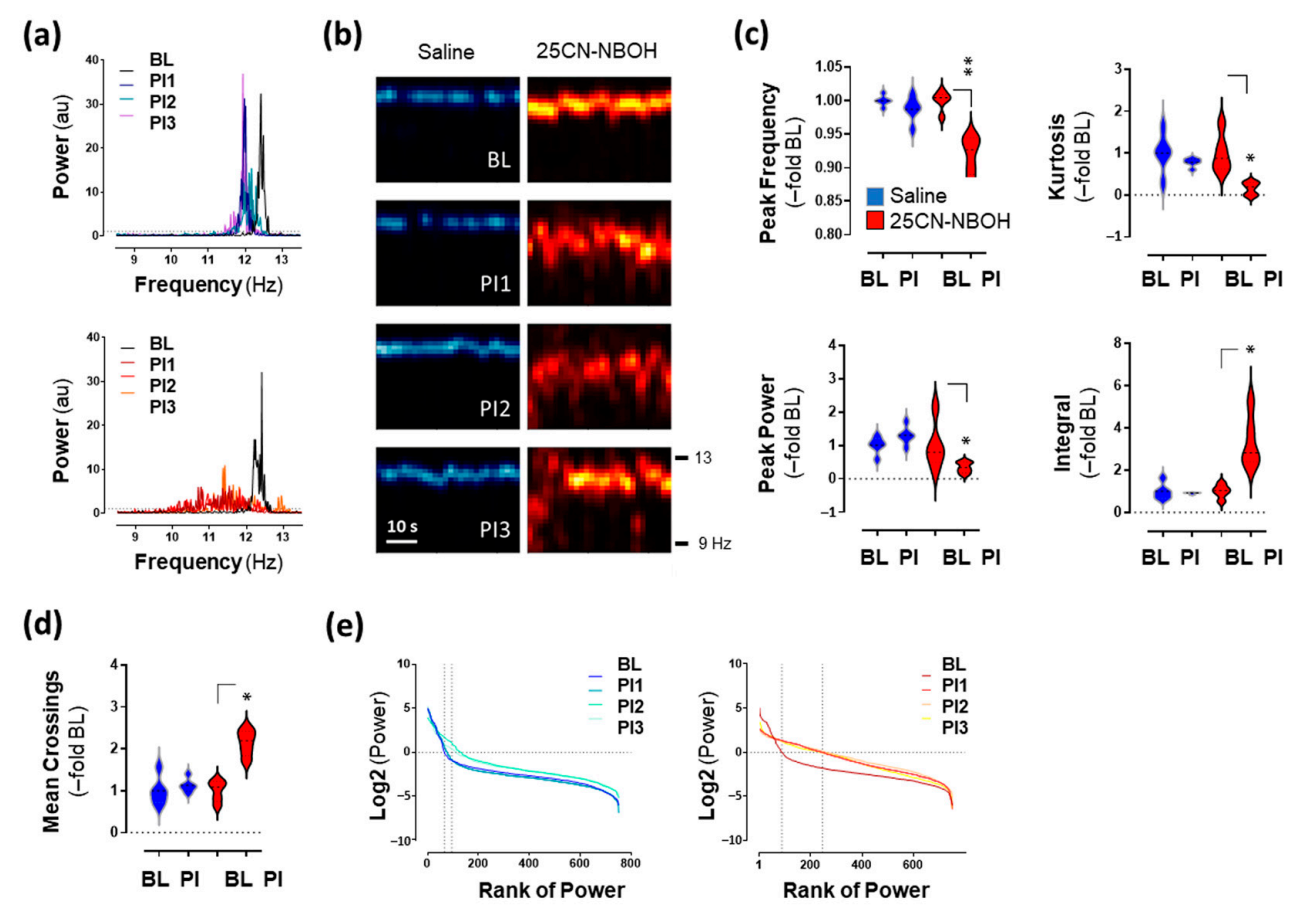
25CN-NBOH disrupts pulsation of arteries above the motor cortex. (**a**) Fourier transform of blood-volume-related signal oscillations (BvSOs) from a representative animal, treated with saline (upper, blue) vs. 1.5 mg/kg 25CN-NBOH i.p. (lower, red) during baseline (BL) and three consecutive recordings post-injection (PI1-3), each 150 s recordings. Au = arbitrary units. (**b**) Spectrogram view of (**a**) (saline: Left, blue; 25CN-NBOH: Right, red; bright color indicates high power). (**c**) Peak-power frequency, peak power, kurtosis, and integral of BvSO Fourier transforms. PI = average of three post-injection recordings. (**d**) Mean-crossings of power-ranked BvSO Fourier transforms. Median and quartiles (–fold BL), ** *p* ≤ 0.01 and * *p* ≤ 0.05. (**e**) Mean-crossings of power-ranked BvSO Fourier transform from a representative animal treated with saline (left, blue) vs. 25CN-NBOH (right, red) during BL and PI1-3. Power values are ranked top to bottom, depicted as Log2, and their mean set at 0 (horizontal line). Vertical lines indicate mean crossings during BL and PI, respectively.

To investigate the effect of 25CN-NBOH on cortical hemodynamics, donor fluorescence sampled across the motor cortex was spectrally analyzed in the heartbeat frequency domain. Baseline (BL) and control condition BvSOs were dominated by a single peak frequency in the 11.5–12.5 Hz range. In the 25CN-NBOH condition, there is an increase in the power of lower power frequencies (Figure 1a,b). This is quantified as a reduction in peak power (median/IQR [–fold BL]: Friedman statistic = 10.68, *p* ≤ 0.01; 1.00/0.79–1.2 to 1.31/1.1–1.5 [control], n.s.; 0.8/0.5–1.6 to 0.34/0.18–0.5 [25CN-NBOH], *p* = 0.05) and peak-power frequency (median/IQR [–fold BL]: Friedman statistic = 12.12, *p* ≤ 0.01; 1.0/0.99–1.0 to 0.99/0.97–1.0 [control], n.s.; 1.0/0.99–1.0 to 0.93/0.89–0.94 [25CN-NBOH], *p* ≤ 0.01). This is also reflected in the features of the shape of the BvSO Fourier transforms (Figure 1c). Whereas the steepness (kurtosis) of the transforms flattened (median/IQR [–fold BL]: Friedman statistic = 9.96, *p* ≤ 0.01; 1.0/0.65–1.35 to 0.78/0.69–0.86 [control], n.s.; 0.88/0.62–1.44 to 0.19/0.00–0.3 [25CN-NBOH], *p* ≤ 0.05), the area-under-the-curve (integral) became larger (median/IQR [–fold BL]: Friedman statistic = 9.24, *p* ≤ 0.01; 1.0/0.68–1.32 to 0.92/0.86–0.93 [control], n.s.; 1.0/0.71–1.3 to 2.83/2.40–4.6 [25CN-NBOH], *p* ≤ 0.05). Similarly, when ranking the power values of the Fourier transforms by their size, 25CN-NBOH increased the number of values excelling noise (mean crossings) (Figure 1d,e) indicating a reduction in the low-to-high asymmetry of the power distribution. No such change was observed for saline treatment (median/IQR [–fold BL]: Friedman statistic = 10.68, *p* ≤ 0.05; 1.0/0.72–1.3 to 1.1/0.99–1.27 [control], n.s.; 1.1/0.74–1.2 to 2.2/1.8–2.4 [25CN-NBOH], each *p* ≤ 0.05).

### 2.2. 25CN-NBOH Did Not Substantially Alter Gross Motor Output

**Figure 2:**
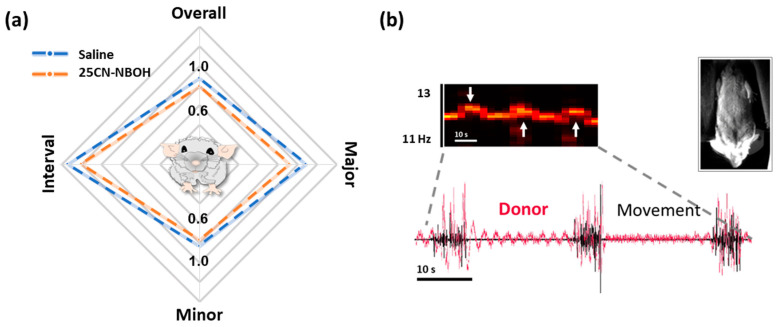
25CN-NBOH does not substantially alter gross mouse motor output. (**a**) Radar chart for overall, major, and minor movements, and temporal distance between individual movements of animals treated with saline (blue) vs. 1.5 mg/kg 25CN-NBOH (red), i.p. Median over three consecutive recordings post-injection, every 150 s (–fold baseline), n.s. (**b**) Ventral-view body movement trace of three representative strong-amplitude movements of saline-treated animals (black) and its effect on the motor cortex donor emission (red). Note: Although strong movements leave a clear mark in the donor spectrogram (upper left inset, white arrows), the spectral pulse signature is maintained. The upper right inset in (**b**) shows a frame of a representative body close-up recording (ventral view).

Next, to test whether the observed effects of 25CN-NBOH on BvSOs are secondary to the gross motor output, body close-up monitoring synchronized to brain imaging was analyzed. There seemed to be an overall tendency of animals to reduce body movements over a recording session (Figure 2a). Statistically, however, this tendency could neither be corroborated for any of the four measurements taken (Friedman statistic = 7.08, 3.12, 7.08, and 4.44, each n.s.). Figure 2b shows a representative movement trace of three high-amplitude movement bursts of a saline-treated mouse, synchronized to the BvSO measured from the motor cortex. Even though the body movements left a clear mark on the donor signal, the donor spectrogram kept its narrow-band structure in frequency. Importantly, these results indicate that the observed hemodynamic responses to 5-HT2A receptor activation are not caused by an overall increase in motor activity.

### 2.3. 25CN-NBOH Induced Shaking Behavior

**Figure 3:**
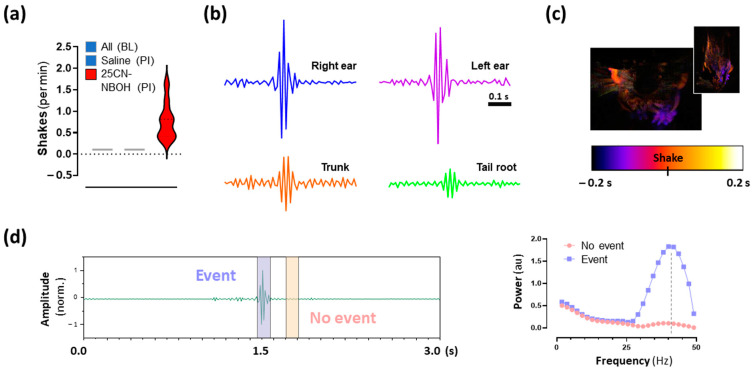
25CN-NBOH induced shaking behavior in mice. (**a**) Number of shaking events per minute during baseline (BL) and three consecutive recordings post-injection (average [PI]), each 150 s recordings, after treatment with saline or 1.5 mg/kg 25CN-NBOH, i.p. median and quartiles. (**b**) Movement traces of a representative shaking event, as measured at ears (upper), trunk, and tail root (lower). (**c**) Color-coded time of event-flanking movements from anterior and ventral perspective, respectively; orange coloring indicates body parts engaged in the shake (see Movie S1 for comparison). (**d**) Sliding window analysis of a representative shaking event (ear ROI) compared to a no-event signal. Note: Shaking peaks in the gamma frequency range (~40 Hz).

Under head fixation conditions, 25CN-NBOH evoked 0.82/0.41–1.02 shaking events per minute (median/IQR, N = 18). No shakes were observed under baseline or after saline treatment (Figure 3a). Pixel-variation traces of different body ROIs revealed that shaking behavior affected the body as a whole and could be observed from the head down to the tail; the most prominent signal was derived from the ears (Figure 3b–d). Individual shakes lasted 0.08/0.07–0.1 s, were temporally separated by 49.4/33.7–73.5 s, and reached their power peak at 42.2/37.5–50.0 Hz (Figure 3d). For a close-up recording of a representative shake, see Movie S1.

### 2.4. Hemodynamic Activity in the Motor Cortex Associated with 25CN-NBOH-Induced Shaking Events

**Figure 4:**
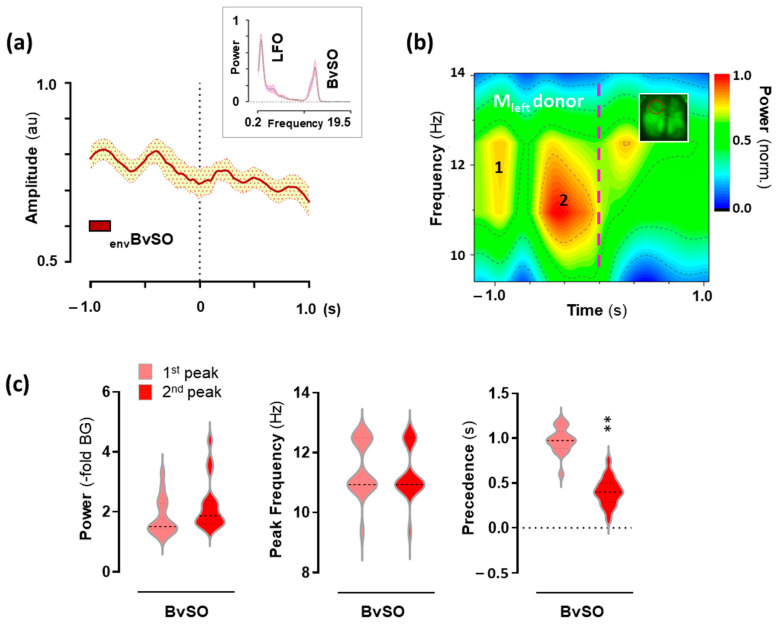
Hemodynamic activity in the motor cortex associated with shaking behavior induced by 25CN-NBOH. (**a**) BvSO activity in the motor cortex, as averaged for their time Fourier transforms (upper inset; frequency in Hz) and event-envelopes in the time domain, respectively. Mean ± SEM. (N = 18 events). (**b**) Mean spectral contour plot of BvSO derived from donor emission. Dashed vertical line denotes time point of peak amplitude of shaking events. Inset shows a mouse brain (dorsal view), red circle denotes the left motor cortex. (**c**) Power increase (–fold background), peak-power frequency (Hz), and temporal precedence (s) of peak 1 and 2 to event, as extracted from the individual spectrograms averaged in (**b**). Median and quartiles, ** *p* ≤ 0.01. BvSO = Blood-volume-related signal oscillations.

To analyze the cortical hemodynamics associated with shaking behavior, BvSOs were extracted in 3-s epochs associated with individual shakes (N = 18 events). BvSOs constitute a distinctive feature of the donor signal and reach LFO comparable power (Figure 4a, upper inset). The averaged signal envelope and the averaged spectrograph of the BvSO epoch Fourier transforms revealed that the shaking behavior was preceded by two BvSO summits (Figure 4a,b), with respective power increases of 1.5/1.4–2.3 and 1.97/1.6–2.4–fold from the background (median/IQR), respective peak frequencies at 10.9/10.9–12.5 and 10.9/10.9–12.5 Hz, and precedence to shaking behavior at 0.97/0.89–1.1 s and 0.4/0.3–0.5 s (Figure 4c). The summits could statistically be separated as to their temporal relation (W = −171, *p* ≤ 0.01), but not as to power or frequency (W = 55 and −12, n.s.).

### 2.5. Low-Frequency Oscillations (LFO) in the Motor Cortex Associated with 25CN-NBOH-Induced Shaking Events

**Figure 5:**
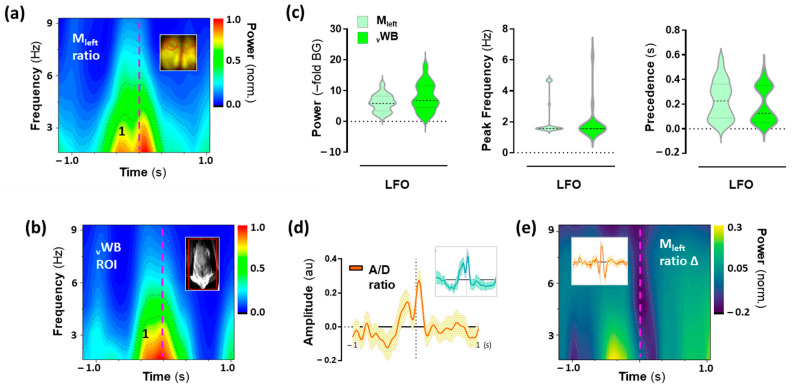
Low-frequency oscillatory (LFO) activity in the motor cortex is associated with shaking events induced by 25CN-NBOH. (**a**) Mean spectral contour plot of LFO activity in the motor cortex. (**b**) Mean spectral contour plot of the whole-body motor output (movements trace, ventral view). Insets show a mouse brain (dorsal view) and a frame of the close-up body recording (ventral view) with areas of recording circled in red. The dashed vertical line denotes the time point of the shaking-behavior peak amplitude. (**c**) Power increase (–fold background), peak-power frequency (Hz), and temporal precedence (s) of peak 1 to shaking behavior, as extracted from the individual spectrograms averaged in (**a**,**b**). Median and quartiles, NS. (**d**) LFO activity in the motor cortex and the movement trace (upper inset), as averaged in the time domain. Mean ± SEM, N = 18 events. (**e**) Mean spectral contour plot of LFO activity in the motor cortex corrected for hemodynamics; upper inset depicts hemodynamics, as averaged in the time domain. A/D = acceptor/donor (ratio); vWB = whole body, ventral view.

Next, to analyze the oscillatory activity of pyramidal neurons associated with the shaking behavior, event-related epochs (N = 18 events) were extracted from the fluorescence signals recorded from pyramidal neurons in the motor cortex and first analyzed for their LFO content. The group-average spectrograph representation of the activity Fourier transform revealed that shaking behavior was embedded in a dominant, bimodal LFO summit (Figure 5a). Its shaking-behavior precedent peak increased by 5.8/3.4–8.2 from the background (BG) (median/IQR), reached its maximum at 1.56/1.56–1.95 Hz, and occurred 0.2/0.1–0.4 s before the motoric execution of shaking behavior (Figure 5c). Analyzing the shaking behavior surrounding pixel-variations in the close-up body recording, a similar LFO-like summit could be detected in the motor output of the animals (Figure 5b), with a power of 6.8/4.5–11.6–fold BG, the maximum frequency of 1.6/1.5–1.5 Hz, and shaking behavior precedence 0.12/0.05–0.35 s (Figure 5c). The features of whole-body motor output were statistically inseparable from the activity in the motor cortex (W = 53, −6, and −37, each n.s.), which is also visible in the time domain (Figure 5d). The first peak of the bimodal summit in the motor cortex reflected voltage changes, and the second was hemodynamics (Figure 5e).

### 2.6. High-Frequency Oscillations (HFO) in the Motor Cortex Associated with 25CN-NBOH-Induced Shaking Events

**Figure 6:**
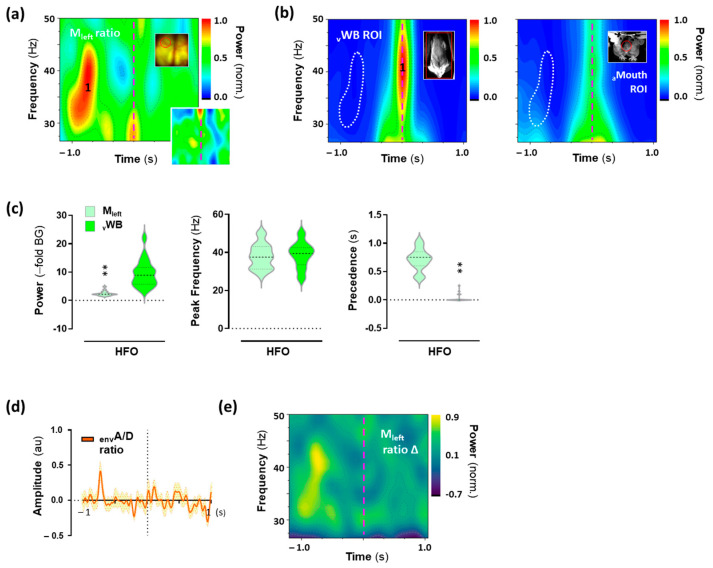
High-frequency oscillatory (HFO) voltage activity in the motor cortex is associated with shaking behavior induced by 25CN-NBOH. Mean spectral contour plot of HFOs (**a**) in the left motor cortex, the right motor cortex (lower inset), and (**b**) in the motor output (movement trace, ventral view [left], and anterior view [right]) (N = 18 events). Upper insets in (**a**,**b**) show a mouse brain (dorsal view) and a frame of the close-up body recording (ventral and anterior view, respectively) with areas of recording circled in red. The dashed vertical line denotes the time point of shaking behavior peak amplitude. Note: At the time-point of the motor-cortical gamma peak, there is nothing within the animals’ motor output that could account for it (white dotted line in (**b**)). (**c**) Power increase (–fold background), peak-power frequency (Hz), and temporal precedence (s) of peak 1 to shaking behavior, as extracted from the individual spectrograms averaged in (**a**,**b**). Median and quartiles, ** *p* ≤ 0.01. (**d**) HFO activity in the motor cortex, as averaged for event envelopes in the time domain. Mean ± SEM (N = 18 events). (**e**) Mean spectral contour plot of HFO activity in the motor cortex corrected for hemodynamics. aMouth = mouth, anterior view; BvSO = Blood-volume-related signal oscillations; envA/D = acceptor/donor, envelope; LFO = Low-frequency oscillations; vWB = whole body, ventral view.

**Figure 7:**
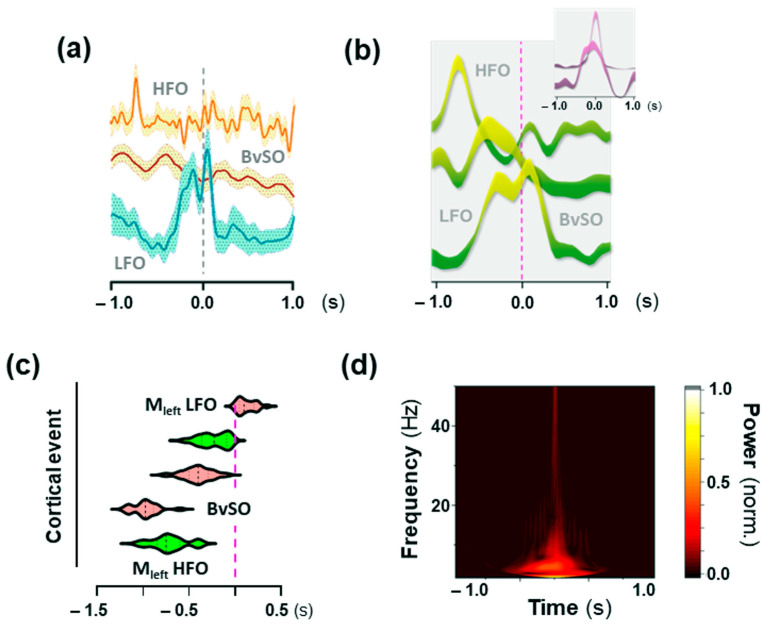
Time-course of cortical events associated with shaking behavior. Oscillatory components detected in the motor cortex are depicted (**a**) in the time domain and (**b**) as peak-power contours in the time-frequency domain (mean ± SEM: Y-scale is arbitrarily offset for visualization). Peak-power contours were extracted from the spectrograms in Figure 4, Figure 5 and Figure 6; upper inset in (**b**) depicts the associated motor output. (**c**) Distribution of oscillatory peaks within the motor cortex relative to the shaking event. Median and quartiles. Green = primarily voltage-related; red = primarily blood-related. (**d**) Fourier transform of a representative shaking event (ear ROI) with the window size fixed in frequency domain. Note: LFO set the motoric platform for HFO. BvSO = Blood-volume-related signal oscillations; HFO = High-frequency oscillations; LFO = Low-frequency oscillations.

Then, for the HFO content of the shaking-behavior-specific cortical activity signals, averaged spectrograms showed that shaking behavior was preceded by a spectrally widespread HFO summit in the motor cortex (Figure 6a,d), which in the right motor cortex appeared to merge with the onset of shaking behavior (Figure 6a, lower inset). The summit’s power increased by 2.2/1.9–2.7 from the background (BG) and peaked at 37.5/31.2–43.2 Hz (median/IQR). It occurred 0.75/0.6–0.85 s before the motoric execution of shaking behavior (Figure 6c), making its precedence highly significant (W = −171, *p* ≤ 0.001). The spectral spread of the HFO summit in the motor cortex closely mirrored the motor execution of shaking behavior, as reflected in the Fourier transform of the whole-body movement signal (Figure 6b). The power increase in behavior was higher than in the motor cortex (median/IQR [–fold BG]: 8.9/5.7–11.7; W = 169, *p* ≤ 0.01). As to their peak frequency, however, both summits were inseparable (Figure 6c) (median/IQR [Hz]: 39.45/33.6–42.6 [whole body, pixel-variation]; W = 8.0, n.s.). At the time-point of the motor-cortical HFO summit, there was no motor output in the whole body or the mouth ROI (Figure 6b). Additionally, when correcting for hemodynamics, the summit could be identified as voltage-related (Figure 6e). Time-course of shaking-behavior-related motorics and cortical motor events is summed up in Figure 7a–d.

## 3. Discussion

5-HT2A receptor activation is the primary molecular substrate of psychedelic action in humans and underlies psychedelic-induced shaking behavior in rodents. Further, non-psychedelic 5-HT2A receptor agonists fail to induce shaking behavior, indicating that shaking behavior may be a readout of a psychedelic-specific active state of the 5-HT2A receptor [9]. Given the relevance of 5-HT2A receptors for both neuronal and cardiovascular functioning, here we sought to investigate the psychedelic-induced shaking behavior, through optical monitoring of both voltage activity patterns of pyramidal neurons in the motor cortex and blood-volume-related corticodynamics.

First, we demonstrated that the selective 5-HT2A receptor agonist 25CN-NBOH reduced the amplitude and frequency of synchronous pulse blood-volume-related signal oscillations (BvSO) in the motor cortex. These reductions were accompanied by a change in the shape of the BvSO frequency spectrum, with low-amplitude frequency components emerging. Unlike its effect on locomotion in freely moving mice [28], 25CN-NBOH did not affect motor activity under head fixation, hence the observed BvSO effect does not appear to be a consequence of the behavioral recruitment of the cardiovascular system. BvSOs reflect blood flow mainly within large-amplitude arteries of the superficial brain tissue rather than parenchymal microcirculation. Psychedelics increase blood pressure [29,30,31], possibly resulting from 5-HT2A receptor-related increase in total peripheral resistance [32]. Consequently, the baroreceptor reflex may counteract the sympathetically driven tachycardia [33,34] thereby potentially affecting brain surface BvSOs. Additionally, thermoregulatory changes [35] and/or interaction with 5-HT2A receptors within the Circle of Willis might play a role [36].

Next, we investigated the brain voltage and hemodynamic activity associated with the shaking behavior induced by 25CN-NBOH. Despite head fixation, shaking events could be readily detected from various body parts. Wavelet and spectral characteristics of shaking events, measured from ROI-specific pixel variations analysis of the behavioral monitoring videos comparable to those quantified by magnometer detection of head-mounted or ear-clipped magnets [37,38] or video-based tracking of a fur-glued fluorescent marker [39]. We then used synchronized fluorescence activity imaging to examine the voltage and hemodynamic activity components in the motor cortex associated with shaking behavior. We detected a bimodal low-frequency oscillation (LFO) peak, which could be attributed to whole-body movements setting the “motoric platform” for the execution of shaking behavior. Shaking behavior has been previously linked to fronto-cortical LFO detected via electroencephalography and local field potential recordings [15,16] which is much more prominent in the striatum than in the motor cortex [16]. The LFO summit in the motor cortex detected here reflects neuro- and hemodynamics and might be a component of cortico-striatal coupling. Additionally, indeed, blood flow oscillations have been shown to follow the spectral characteristics of neuronal oscillations [40].

In addition, we also observed a dominant high-frequency oscillation (HFO) summit in the motor cortex that closely mirrored the spectral characteristics of the motoric execution of shaking behavior but preceded the motor action by ~700 ms. Cortical layer 2/3 pyramidal neurons in the frontal and motor cortices express 5-HT2A receptors [41,42,43] and may be directly depolarized by psychedelics or indirectly inhibited through GABAergic interneurons [21]. Layer 2/3 pyramidal neurons innervate the striatum [44] and drive corticospinal layer 5 pyramidal neurons [19,45]. This top-down control may be in addition to the direct action of psychedelics on layer 5 pyramidal neurons [12] and facilitate smooth coordination of shaking behavior with other motor output. A role in coordination, rather than execution, would also align with results indicating that shaking behavior may also be executable without the motor cortex [13,14]. Importantly, our results do not exclude the possibility that shaking behavior-relevant 5-HT2A receptors are also localized outside the motor cortex or that the oscillatory components detected may be integrative to processes outside of layer 2/3. Future research, using fiber photometry, projection-specific neuronal silencing, and/or caged ligand activation might help to further clarify the circuits involved.

Further, we found that shaking behavior is preceded by hemodynamic BvSO activity, which (in addition to the factors discussed above) may be driven by pre-event arousal of the heart, local diameter changes in brain-surface arteries [46], and/or the meeting of cortical energy demands. Despite the well-known role of 5-HT2A receptors in cardiovascular functioning [26,47], only limited research has been devoted to a possible disentanglement of vascular and neuronal constituents of psychedelic drug action [48,49]. A future combination of voltage imaging with techniques such as pulse oximetry [35], functional near-infrared spectroscopy [50], and/or laser speckle contrast imaging [51] might help and tell both constituents apart.

## 4. Materials and Methods

### 4.1. Animals and Target Gene Expression

Animals (Ai78 (TITL-VSFPB1.2); Rasgrf2-dCre;CaMK2A-tTA, Jackson Labs (Bar Harbor, MA, USA) [52]) were housed in individually ventilated cages with a 12:12 h light/dark cycle and maintained at an ambient temperature of 21 ± 2 °C at 55 ± 10% humidity. Mice were provided with standard rodent-chow pellets (Special Diet Services, #RM1) and water ad libitum. The transgene encoding the genetically encoded voltage indicator VSFP Butterfly 1.2 was intersectionally expressed in layer 2/3 pyramidal neurons through a non-invasive, transgenic breeding approach (Figure S1a), with indicator expression doubly regulated by Cre recombinase and tTA transactivator. The line is maintained in-house and triple transgene expression verified by PCR. Expression selectivity has been demonstrated by immunohistochemistry [53,54].

### 4.2. Surgery and Experimental Habituation

Cranial window preparation was performed on triple transgenic mice as detailed previously [55,56]. In brief, adult mice were surgically anesthetized with pentobarbital (40–90 mg/kg, i.p.) and implanted with a light-weight metal plate secured to the skull by adhesive resin cement (Super-Bond C&B, Sun Medical, Shiga, Japan). The skull was thinned to create an optical window and reinforced by a layer of clear nail polish. Animals were singly housed after surgery, allowed to recover for at least 7 days, and then habituated to gradually increasing periods of head fixation under the optical imaging macroscope. All experiments were performed with fully awake, habituated animals with a minimum of three days free of anesthetic.

### 4.3. Treatment and Selection

Psychedelics are generally rather unselective drugs and some of their off-targets [57], including several monoamine receptor subtypes, are known to interfere with shaking behavior and/or the electrophysiological response of the cortex to psychedelics [12]. To reduce pharmacodynamic noise unrelated to shaking behavior, we used (N-(2-hydroxybenzyl)-2,5-dimethoxy-4-cyanophenylethylamine (25CN-NBOH)—an agonist with improved selectivity for 5-HT2A receptors [58]. 25CN-NBOH (a kind gift from Jesper L. Kristensen, University of Copenhagen) was dissolved in isotonic saline and administered intraperitoneally (i.p. <10 mL/kg). We administered 25CN-NBOH at 1.5 mg/kg, the optimal dose for 5-HT2A-related behavioral and cardiovascular effects [28,35]. For intrasubject comparison, mice were administered with either saline or 25CN-NBOH on different occasions. For analysis, shaking-behavior active mice were selected (n = 7 with 4 × 7 datasets [within-group controlled]).

### 4.4. Voltage Imaging and Behavioral Monitoring

VSFP Butterfly 1.2 is optimal for chronic in vivo imaging, with only negligible loss of fluorescence (bleaching) over time [59], so animals could be imaged repeatedly. For each imaging session, animals were head-fixed in a custom-made holding bracket, and voltage activity was imaged across both dorsal cortical hemispheres in four trials, each lasting for 150 s. One trial was carried out at 5 min before drug or saline administration (baseline; BL), and three consecutive trials began at 5 min, 13 min, and 21 min after administration (post-injection; PI1-3). Images were acquired at 100 Hz with a dual emission widefield epifluorescence macroscope, using two synchronized CMOS cameras in global shutter mode (Basler AG, Ahrensburg, Germany). Fluorescence excitation was provided using a high-power (150 W) halogen lamp (Moritex, BrainVision, Tokyo, Japan) and the following optics (Semrock, Rochester, NY, USA): Optical filters: 500/24 nm for mCitrine (donor) excitation, FF01-542/27 for mCitrine emissions; BLP01-594R-25 for mKate2 (acceptor) emissions; beam splitter: 515LP for excitation, 580LP for emission detection. Voltage imaging was accompanied by synchronized close-up body video recordings (100 Hz), with cameras positioned rostrally and ventrally to the mice. The animals sat on a transparent acrylic plate allowing for whole-body recording from below (ventral view). Infrared light was used for body illumination to prevent visible-light-related arousal and interference with fluorescence imaging.

### 4.5. Analysis of Motor Behavior

For evaluation of overall motor output, body close-up monitoring videos were analyzed for movement-related intensity (grey level) changes across defined regions of interest (ROIs) using ImageJ software 1.51k (NIH; Bethesda, MD, USA). Intensity traces were derived from the ventral view of the whole body (WB), pre-processed to remove baseline shifts and shot noise, smoothed using a moving average, and analyzed for deflections as indicators of movements. Peaks ≥ 1 SD were designated as major movements, and peaks < 1 SD as minor. Movement frequency was calculated from the mean interval between movement events. The detected post-injection results were averaged and depicted as an x–fold change to the baseline. For analysis of shaking behavior-related motor output, epochs were extracted from the intensity traces obtained from manually selected regions of interest (ROI), including the ears, the mouth, the trunk, the tail, and/or the whole body. The duration of individual shakes (in seconds) was verified by visual inspection of the videos and shake distance was defined as the time between individual shakes (in seconds). Low-frequency components (LFO) and high-frequency components (HFO) of motor output were separated by band-pass filtering (0.5 to 8.0 Hz for LFO, 30 to 50 Hz for HFO), and the frequency of shaking behavior was determined by sliding-window analysis. For power spectra comparison with voltage activity from the motor cortex, epochs of detected movements were normalized, zero-padded, Hann-windowed, and transformed into time x frequency domain using Fourier transformation (with equally spaced, overlapping segments; length: 48 p; offset: 5 p). Power peaks at defined frequency bands were quantified as x–fold changes to the background. Signal processing and analysis were carried out using Sigview 6.0 Pro (SignalLab e.k. [Pforzheim, Germany]) and OriginPro v2023 (OriginLab Corporation, Northampton, MA, USA).

### 4.6. VSFP Butterfly 1.2 Donor Imaging for Reading out Blood Volume

In VSFP Butterfly 1.2, the fluorescent protein mCitrine acts as a FRET donor to generate the voltage-dependent FRET signal. At the same time, mCitrine emission reports the absorption of blue/green light by the blood hemoglobin in the brain tissue, making the mCitrine (donor) emission channel a suitable readout of hemodynamics [20]. The heartbeat frequency band (8.5–13.5 Hz) of the donor signal was analyzed to evaluate vascular effects. These blood-volume-related signal oscillations (BvSO) were normalized, zero-padded, Hann-windowed, and Fourier transformed into the frequency domain. The power distribution was evaluated for peak frequency, peak power, kurtosis, and area under the curve (integral). The low-to-high asymmetry of the power distribution was quantified with values ranked top-down as the number of values crossing spectral noise (set as 0). Post-injection results were averaged and scaled as an x–fold baseline. For time x frequency analysis, BvSO epochs surrounding a shaking event were processed as described for the motor output.

### 4.7. VSFP Butterfly 1.2 Voltage Imaging

Voltage signals from layer 2/3 pyramidal neurons in the motor cortex were calculated as the ratio of gain equalized acceptor-to-donor (A/D) fluorescence (for the mechanism of action of Butterfly 1.2 see Figure S1b) [20]. The gain equalization and ratioing remove the dominant hemodynamic signal of the isolated fluorescence emission channels and thereby extracts the voltage-dependent signal of VSFP Butterfly 1.2. For time x frequency analysis, voltage signal epochs surrounding a shaking event were processed as described for the motor output, and spectrally compared to oscillatory events detected in behavior. For the time-domain depiction of LFO and HFO components, normalized epochs and their envelopes were averaged, respectively.

### 4.8. Statistics

Statistical analysis and graphing were performed with GraphPad Prism (GraphPad Software v9.5 [San Diego, CA, USA]) and OriginPro v2023. Baseline vs. post-injection differences in blood-volume-related signal oscillations and gross motor output were evaluated using a non-parametric Friedman test for dependent samples, followed by a Dunn-corrected post hoc comparison. For the confidence of spectral peaks, a Siegel test was applied (Sigview 6.0 Pro), with epoch-extracted parameters followed up on with the Wilcoxon procedure. Significance was assumed at a probability level of 0.05.

## 5. Conclusions

Given the overwhelming focus on the role of layer 5 pyramidal neurons in psychedelic-induced cortical activity, layer 2/3 pyramidal neurons are largely overlooked despite being a prominent 5-HT2A receptor-expressing population with a crucial role in the execution of top-down control that governs motor output and consciousness. Here, we report a set of activity correlates of psychedelic-induced shaking behavior in the motor cortex. In particular, we highlight (1) the importance of layer 2/3 pyramidal voltage activity as a potential modulatory or integration hub of psychedelic-induced motor output, as well as (2) an impact of selective 5-HT2A agonism on cranial artery pulsation.

## Data Availability

This paper does not report the original code. Any additional information required to analyze the data reported in this paper is available from the lead contact upon request.

## Supplementary Materials

The following supporting information can be downloaded at: https://www.mdpi.com/article/10.3390/ijms24119463/s1.
