# Supplementary material for: Cortical Correlates of Psychedelic-Induced Shaking Behavior Revealed by Voltage Imaging
Source: Int J Mol Sci. 2023 May 30;24(11):9463. doi: 10.3390/ijms24119463 (PMC10253917; doi:10.3390/ijms24119463)
Supplement: Supplementary file 1 [file ijms-24-09463-s001.zip › Figure S1.pdf]

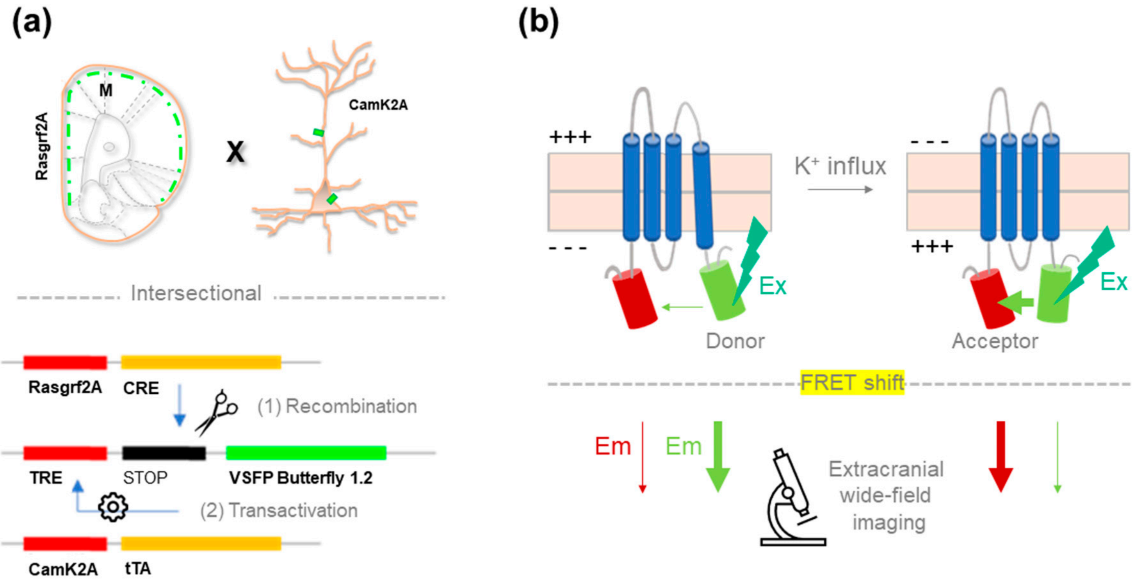

Figure S1. Schematic diagram of VSFP Butterfly 1.2 expression and mechanism. (a) Expression of CRE recombinase removes the STOP cassette in the indicator transgene; this CRE recombinase expression is limited to cortical layer 2/3 as controlled by Rasgrf2A promoter. tTA expression controlled by CamK2A promoter is limited to pyramidal neurons and activates the TRE promoter in these cells. Together, tTA and CRE recombinase expression intersectionally restricts VSFP expression in layer 2/3 pyramidal neurons [53,54]. (b) VSFP Butterfly 1.2 contains a voltage-sensing element (blue) flanked by a pair of green and red fluorescent proteins, and reports fluctuations in membrane voltage as intensity changes in green and red fluorescence emission [20]. Depicted: Ex-citatory synaptic event. CamK2A=Calcium/Calmodulin Dependent Protein Kinase II Alpha; CRE=Cyclization recombinase; Em/Ex=Emission/excitation; FRET=Förster resonance energy transfer; M=Motor cortex; Rasgrf2A=Ras protein-specific guanine nucleotide-releasing factor 2A; tTA=Tetracycline-controlled transactivator; TRE=Tetracycline response element; VSFP=Voltage-sensitive fluorescent protein.

Movie S1. Frontal close-up recording of a 25CN-NBOH induced shaking event. Shaking event induced by a 1.5-mg/kg i.p. dose of 25CN-NBOH (anterior perspective [3.00-s window; recorded at 100 Hz, playing at 10 Hz]). Upper panel, left: Original video; upper panel, right: Video with sta-tionarities subtracted; lower panel: Pixel dynamical change of right ear ROI across time (high-pass filtered). Zero (0:00 s) marks onset of shake. Format: AVI. Duration: 30 s.
